# The Risk of Nosocomial Transmission of Rift Valley Fever

**DOI:** 10.1371/journal.pntd.0004314

**Published:** 2015-12-22

**Authors:** Nasser A. Al-Hamdan, Anil A. Panackal, Tami H. Al Bassam, Abdullah Alrabea, Mohammed Al Hazmi, Yagoub Al Mazroa, Mohammed Al Jefri, Ali S. Khan, Thomas G. Ksiazek

**Affiliations:** 1 Faculty of Medicine, King Fahad Medical City, King Saud bin Abdulaziz University for Health Sciences, Riyadh, Kingdom of Saudi Arabia; 2 United States Public Health Service (USPHS) at the Centers for Disease Control and Prevention (CDC) in Atlanta, Georgia, United States of America; 3 Hafr Albatin Health Region, Hafr Albatin, Kingdom of Saudi Arabia; 4 College of Medicine, Imam University, Riyadh, Kingdom of Saudi Arabia; 5 King Fahad Central Hospital, Jazan, Kingdom of Saudi Arabia; 6 Health Services Council, Ministry of Health, Riyadh, Kingdom of Saudi Arabia; 7 Preventive Medicine Department, Ministry of Health, Riyadh, Kingdom of Saudi Arabia; 8 College of Public Health at University of Nebraska Medical Center, Omaha, Nebraska, United States of America; 9 Galveston National Laboratory, Department of Pathology, University of Texas Medical Branch, Galveston, Texas, United States of America; Centers for Disease Control and Prevention, UNITED STATES

## Abstract

In 2000, we investigated the Rift Valley fever (RVF) outbreak on the Arabian Peninsula—the first outside Africa—and the risk of nosocomial transmission. In a cross-sectional design, during the peak of the epidemic at its epicenter, we found four (0.6%) of 703 healthcare workers (HCWs) IgM seropositive but all with only community-associated exposures. Standard precautions are sufficient for HCWs exposed to known RVF patients, in contrast to other viral hemorrhagic fevers (VHF) such as Ebola virus disease (EVD) in which the route of transmission differs. Suspected VHF in which the etiology is uncertain should be initially managed with the most cautious infection control measures.

## Author Summary

Rift Valley fever (RVF) is a zoonosis that is primarily transmitted to humans via infected mosquito bites. Although most acute RVF infection results in a nonspecific febrile illness, 1% of cases develop hemorrhage and 50% of these may result in death. Person-to-person transmission has not been described, but high viral titers have been observed during hemorrhagic complications, suggesting the potential for direct person-to-person transmission. This article describes an estimated risk of RVF nosocomial transmission during an outbreak setting and contrasts the suggested infection control precautions to that of other viral hemorrhagic fevers (VHF) such as Ebola virus disease (EVD).

## Introduction

Rift Valley fever (RVF) is a zoonotic disease caused by an RNA virus in the genus *Phlebovirus*, family Bunyaviridae. RVF virus is transmitted to humans primarily by mosquito bites and by direct contact with infected animal body fluids [1]. First described in Kenya in 1910, the disease has been recognized in many African countries with a severity ranging from localized, well controlled clusters to major epizootics and associated epidemics [2]. In August 2000, the first confirmed occurrence of RVF outside the African continent was described on the Arabian Peninsula along the Red Sea coast in southwestern Saudi Arabia and Yemen. This outbreak illustrated that the RVF virus can adapt to different ecological conditions and cause infection in humans and domestic ungulates, provided suitable mosquito vectors and animal reservoirs are present.

Although most acute RVF virus infections result in a nonspecific febrile illness, the virus is hepatotrophic and associated with hepatitis, and a concomitant nephropathy has been described [3]. In addition, 1% of cases develop hemorrhagic complications and up to 50% of these may result in death. Encephalitis may occur in 1% or more of cases 1 to 4 weeks after the acute illness resolves (Available via CDC at: http://www.cdc.gov/vhf/rvf/RVF-FactSheet.pdf; available via WHO at: http://www.who.int/mediacentre/factsheets/fs207/en/) [4]. During the first 4 weeks after recovery, as many as 15% of cases may result in ocular complications, such as retinitis, and up to 50% may have permanent vision loss [5–7].

Person-to-person transmission has not been described, but laboratory workers are known to be at risk for RVF virus infection possibly, via aerosolization [4]. Human infection readily occurs from contact with infected animal blood and amniotic fluid, in which RVF virus has been reported to reach titers of 10^10^ virions per ml [8]. Similar titers, 10^8^ among infected humans, who may develop frank hemorrhage, have suggested the possibility that direct person-to-person transmission may occur [9]. However, the true risk to health-care workers (HCWs) for acquiring RVF in the hospital setting is unknown. To estimate the magnitude of such a risk, we undertook a descriptive observational cross-sectional study to evaluate nosocomial acquisition of RVF in Jazan, where protective measures were promulgated to hospitals admitting RVF cases.

## Methods

The study was conducted under the auspices of the Ministry of Health and Field Epidemiology Training Program, Kingdom of Saudi Arabia and with the assistance of CDC as an outbreak response related activity. In addition, we obtained visiting country equivalent institutional review board (IRB) approval for a clinical trial of ribavirin for RVF as an adjunct to this study–all part of the overall RVF outbreak response. The risk to HCWs for acquiring RVF in the hospital setting was assessed at four hospitals in the Jazan province–where the outbreak began—during October 22–26, 2000, which corresponded to the end of the peak of the outbreak (three months after it began in August 2000): King Fahad Central Hospital (KFCH), Samtah General Hospital (SGH), Al Ardah Hospital (AH), and Beash Hospital (BH). KFCH was the regional referral hospital, whereas the others were located in the hyperendemic areas. The study was begun approximately three months into the RVF outbreak in Jazan, when on average 50 to 75 new cases were being reported on a weekly basis. From August to October, a total of approximately 400 RVF patients were hospitalized at these four facilities. We were not able to obtain information on how many required intensive care unit admission or had severe manifestations, but these likely represented the minority, given what is known about the natural history of most RVF infections.

A cross-sectional cohort from each hospital was selected of approximately 50–150 HCWs who were in close contact with 10 or more RVF patients, their body fluids, or other potentially infectious materials with RVF virus. These cohorts were composed of individuals such as laboratory technicians, phlebotomists, morgue workers, physicians, nurses, orderlies, and cleaning staff working in areas of the hospital with RVF patients and/or potentially contaminated materials (“high-risk group”). Such persons were most likely to have bloodborne pathogen exposure via needlestick, mucosal splashes, or large mucocutaneous exposures to blood or tissue from infected individuals. Another set of cohorts of approximately 50–150 HCWs who did not have much, if any, such exposure was also chosen from the same hospitals; this group included individuals working primarily in the areas of pediatrics, obstetrics and gynecology, psychiatry, pharmacy, social work, and hospital administration (“low-risk group”). Trained interviewers administered a questionnaire to the HCWs in both groups to collect information about their demographics, level and type of hospital exposures, precautionary measures, and possible environmental exposures. All enrolled HCWs were assigned a unique code number designed to assist efforts to accurately identify the specimens and ensure confidentiality.

A blood sample (5 ml) was taken from each participant to test for IgM and IgG antibody to RVF virus, using an enzyme-linked immunosorbent assay (ELISA). Antibodies to RVF virus were detected by using both IgM and IgG assays and inactivated RVF virus antigens. Both were done by using methods previously described for Ebola virus [10]. Briefly, the IgM assay was performed in a Mu-capture format using RVF antigen grown in Vero E6 cells and inactivated by gamma irradiation and a hyperimmune anti-RVF mouse ascitic fluid as the detection system for bound antigen. The IgG assay employed a detergent-extracted RVF antigen grown in Vero E6 cells and inactivated by Gamma irradiation; antigen was adsorbed directly onto microtiter plates. Both IgG and IgM assays were performed using mock-infected Vero E6 antigens prepared in the same manner, respectively. Sera were tested at dilutions from 1:100 to 1:6400 in 4-fold dilutions. Samples were considered positive for the IgM assay if 1) the sum of the adjusted optical densities from all of the dilutions (infected antigen less the mock infected antigen) was greater than 0.45 through the entire dilution series and 2) the titer was 1:400. Samples were likewise considered positive in the IgG assay if 1) the sum for the adjusted optical densities from all of the dilutions (infected antigen less the mock infected antigen) was greater than 0.95 through the entire dilution series and 2) the titer was 1:400. The IgM-capture assay employed goat anti-human Mu to capture IgM (Biosource, Camarilla, CA) and a horseradish peroxidase conjugated goat anti-mouse IgG from Biosource in the RVF antigen detection system. The IgG assay used a horseradish peroxidase conjugated mouse anti-human Gamma-chain-specific antibody (Accurate Chemical, Westbury, NY) to detect bound IgG. These tests were performed at the National Polio Laboratory (Riyadh, Kingdom of Saudi Arabia) and confirmed by the Centers for Disease Control and Prevention (Atlanta, GA, USA). Evidence of infection during the epidemic was defined as any individual in the cohort with detectable IgM and IgG antibody to RVF virus. This assessment was performed as a public health emergency declared by the Saudi Ministry of Health and carried out at their urgent request for institutional assistance to assess infection control practices.

## Results

A total of 703 HCWs participated in this study. Three hundred and forty-six (49%) were males and the mean age was 33 years (range: 20–64 years; standard deviation: ± 9 years). The most common nationalities included Indians (37%), Saudi Arabians (26%), and Filipinos (12.5%). Two hundred sixty-six (37.8%) were from KFCH and 240 (34.1%) were from SGH, where precautionary measures, such as the use of gloves, gowns, and face masks, were widely implemented. However, of the remaining HCWs, one hundred eleven (15.8%) were from AH and 86 (12.2%) were from BH, where the use of protective measures was less common. By occupation, 80 (11%) were physicians, 312 (44.6%) were nurses, 43(6.2%) were laboratory technicians, 115 (16.5%) were cleaners, and 153 (21.7%) had other jobs.

With respect to community exposure, 74 (10.7%) of the 703 participants reported direct contact with animals; among these, 15 (20.3%) reported that they slaughtered animals between August and October 2000. Of the 703, 42 and 57 (6.0 and 8.1%) reported a history of exposure to animal abortions or deaths, respectively, around their residence. Mosquitoes were reportedly present at the residence of 347(49%) participants, but only 242 (35%) participants reported having had mosquito bites.

Two hundred sixteen (29%) participants reported contact with 10 or more RVF patients. Twenty (2.9%) of the 685 HCWs who completed this item on the questionnaire reported having needlesticks from suspected or confirmed RVF patients within the prior 2 months; up to 57 (9%) reported some unprotected exposure to various body fluids (e.g. blood, urine, feces, spinal fluid, and saliva) from suspected or confirmed RVF patients during that same period. Unfortunately, HCW did not recall details of the severity of percutaneous or mucosal exposure to blood or tissue from RVF patients, as parameters such as volume and viral load of occupational exposure has been reported to increase the risk of transmission with other bloodborne viruses such as HIV. Despite these potentially “high-risk” nosocomial exposures, none of these HCWs were found to have evidence of infection with RVF virus.

With respect to the self-reported protective measures employed by HCWs, 72.1% reported always wearing gloves, 68% face masks, and 60.8% gowns when they dealt with suspected or confirmed RVF patients, body fluids, or material (Table 1).

**Table 1:** Self-reported use of protective measures by health-care workers (HCWs) (N = 703) when working with RVF patients in 4 Jazan hospitals. Jazan, October 22–26, 2000.

Protective Measure	No. of users	Percent
Glove	486	72.1
Face-mask	457	68
Gown	404	60.8
Head cover	322	48
Foot cover	278	41.6
Eye protection	114	17.1

Four (0.6%) of 703 HCWs had evidence of recent RVF virus infection as indicated by IgM positivity. All four reported no known or no contact with patients with suspected or confirmed RVF; all reported no needlestick exposure or direct contact with body fluids from RVF patients. All RVF antibody positive HCWs were in the “low-risk group.” Three worked at AH and one at KFCH. Two were orderlies who worked in the medical ward and intensive care unit, 1 was a security guard, and 1 was a clerk. Three reported having a febrile illness in the past 2 months. All four RVF-antibody-positive HCWs reported having mosquitoes at their place of residence; the number of bites ranged from “sometimes” to “frequently.” All three HCWs from AH reported close contact with animals; of these, two also reported exposure to dead or aborted animals. Of note, Al Ardah was the area where the first RVF cases and the greatest number of cases in Jazan were reported, and where a 90% antibody prevalence ratio was identified among animals in a survey done in this area [2]. None of the HCWs were IgG positive at the time of the study. Moreover, despite 40% of staff not using contact precautions and the 100% not following airborne/droplet precautions, RVF seroconversion did not occur. Therefore, we infer that standard precautions would suffice in managing RVF patients.

## Discussion

Serological evidence suggests that only four (0.6%) of the 703 HCWs were infected by RVF virus. Our data suggest that these infections were probably the result of community exposure rather than nosocomial acquisition. Nosocomial transmission, if it occurs, appears to be very rare in the context of at least rudimentary standard precautions. These data suggest that the risk for hospital-acquired RVF in HCWs is very low and that the use of standard precautions alone afford sufficient protection to HCWs who deal with known RVF patients.

Strengths of this study included high-risk and low-risk groups being well-defined and data acquired during the end of the peak of the epidemic, within its epicenter, at a regional referral hospital and 3 hospitals in the surrounding hyper-endemic community in order to minimize ascertainment bias. The study target size was large and extrapolated from the number of cases occurring per week at the time of the study in the Jazan province.

However, with the recent Ebola virus disease (EVD) epidemic of 2014 –the largest to date, centered in West Africa–caution is advisable in those with suspected viral hemorrhagic fever (VHF).[11] VHF syndromes among the various etiologies can overlap, though the infectiousness and route of transmission may differ considerably. EVD and RVF exemplify this. This EVD outbreak appears to have been caused by Zaire ebola virus, originally identified as the etiology in the 1976 Democratic Republic of the Congo (DRC) outbreak that had a case fatality proportion of approximately 90%, and similarly began in rural forest communities [12–14]. In contrast, the 2014 epidemic has had a case fatality proportion of approximately 60–70%.While standard, contact, and droplet precautions are recommended for management of hospitalized patients with known or suspected EVD (Available at: http://www.cdc.gov/vhf/ebola/hcp/infection-prevention-and-control-recommendations.html) [15], our study found that standard precautions alone may suffice for RVF. Given the established high nosocomial transmissibility via body fluids of EVD, while personal protective equipment (PPE) for EVD is extensive, including single-use (disposable) fluid-resistant gowns that extend to at least mid-calf with single-use “double gloving” and full face shield/facemask (Available at: http://www.cdc.gov/vhf/ebola/healthcare-us/ppe/guidance.html), such PPE appears unnecessary for RVF based upon our study findings. Since the geographical and syndromic distribution of EVD and RVF may coincide, particularly with the frequency of air travel, isolation of individuals suspected to have symptoms of VHF of undetermined etiology seems prudent, adopting the most conservative barrier methods pending the etiologic diagnosis. Co-infection of EVD and RVF is also a possibility. Fortunately, bunyaviruses such as RVF virus and filoviruses such as Ebola are sufficiently different that current serodiagnostic methods should have a high discriminatory index, unlike alphaviruses and flaviviruses.[16] Future studies on rapid diagnostics that shorten the pre-patent in relation to the incubation period and are etiology specific will be invaluable to curb the spread of these deadly VHFs.

## Supporting Information

S1 Checklist STROBE (STrengthening the Reporting of OBservational studies in Epidemiology) checklist provides annotation of the elements fulfilled in this article as outlined by the international, collaborative initiative of epidemiologists, methodologists, statisticians, researchers and journal editors involved in the conduct and dissemination of observational studies to enhance quality. (DOC)
