# Supplementary material for: The Risk of Nosocomial Transmission of Rift Valley Fever
Source: PLoS Negl Trop Dis. 2015 Dec 22;9(12):e0004314. doi: 10.1371/journal.pntd.0004314 (PMC4687845; doi:10.1371/journal.pntd.0004314)
Supplement: S1 Checklist — (DOC) [file pntd.0004314.s001.doc]

STROBE Statement—Checklist of items that should be included in reports of ***cross-sectional studies***

|  | Item No | Recommendation |
| --- | --- | --- |
| **Title and abstract** | 1 | (*a*) Indicate the study’s design with a commonly used term in the title or the abstract  **Cross-sectional design is listed in the revised abstract** |
|  |  | (*b*) Provide in the abstract an informative and balanced summary of what was done and what was found  **In 2000, we investigated the Rift Valley fever (RVF) outbreak on the Arabian Peninsula -- the first outside Africa -- and the risk of nosocomial transmission. In a cross-sectional design, during the peak of the epidemic at its epicenter, we found four (0.6%) of 703 healthcare workers (HCWs) IgM seropositive but all with only community-associated exposures. Standard precautions are sufficient for HCWs exposed to known RVF patients in contrast to other viral hemorrhagic fevers (VHF) such as Ebola virus disease (EVD) in which the route of transmission differs. Suspected VHF in which the etiology is uncertain should be initially managed with the most cautious infection control measures.** |
| Introduction | | |
| Background/rationale | 2 | Explain the scientific background and rationale for the investigation being reported  **Page 6 of revised cleaned version, lines 1-9.** |
| Objectives | 3 | State specific objectives, including any prespecified hypotheses  **Page 6 of revised cleaned version, lines 7-9 lists the objective** |
| Methods | | |
| Study design | 4 | Present key elements of study design early in the paper  **Page 6 of revised cleaned version, lines 12-22 and page 7** |
| Setting | 5 | Describe the setting, locations, and relevant dates, including periods of recruitment, exposure, follow-up, and data collection  **Page 6 of revised cleaned version, lines 16-22 and page 7, lines 1-2** |
| Participants | 6 | (*a*) Give the eligibility criteria, and the sources and methods of selection of participants  **Page 6 of revised cleaned version, lines 6-16** |
| Variables | 7 | Clearly define all outcomes, exposures, predictors, potential confounders, and effect modifiers. Give diagnostic criteria, if applicable  **Page 7 of revised cleaned version, lines 7-20** |
| Data sources/ measurement | 8* | For each variable of interest, give sources of data and details of methods of assessment (measurement). Describe comparability of assessment methods if there is more than one group  **Page 7 of revised cleaned version, lines 7-20 and page 8. Study was primarily descriptive and healthcare worker cross-sectional seroprevalence study.** |
| Bias | 9 | Describe any efforts to address potential sources of bias  **High-risk and low-risk groups were defined and data acquired during the end of the peak of the epidemic at a regional referral hospital and 3 hospitals in the surrounding hyper-endemic community to minimize ascertainment bias.** |
| Study size | 10 | Explain how the study size was arrived at **Extrapolated from the number of cases occurring per week at the time of the study in the Jazan province** |
| Quantitative variables | 11 | Explain how quantitative variables were handled in the analyses. If applicable, describe which groupings were chosen and why **Descriptive epidemiology and seroprevalence only** |
| Statistical methods | 12 | (*a*) Describe all statistical methods, including those used to control for confounding **N/A** |
|  |  | (*b*) Describe any methods used to examine subgroups and interactions **N/A** |
|  |  | (*c*) Explain how missing data were addressed **N/A** |
|  |  | (*d*) If applicable, describe analytical methods taking account of sampling strategy **N/A** |
|  |  | (*e*) Describe any sensitivity analyses **N/A** |
| Results | | |
| Participants | 13* | (a) Report numbers of individuals at each stage of study—eg numbers potentially eligible, examined for eligibility, confirmed eligible, included in the study, completing follow-up, and analysed **page 9 of revised cleaned version, lines 5-20, page 10, and page 11, lines 1-7** |
|  |  | (b) Give reasons for non-participation at each stage **N/A** |
|  |  | (c) Consider use of a flow diagram **N/A** |
| Descriptive data | 14* | (a) Give characteristics of study participants (eg demographic, clinical, social) and information on exposures and potential confounders **page 9 of revised cleaned version, lines 5-20, page 10, and page 11, lines 1-7 and Table 1** |
|  |  | (b) Indicate number of participants with missing data for each variable of interest **N/A** |
| Outcome data | 15* | Report numbers of outcome events or summary measures **page 9 of revised cleaned version, lines 5-20, page 10, and page 11, lines 1-7** |
| Main results | 16 | (*a*) Give unadjusted estimates and, if applicable, confounder-adjusted estimates and their precision (eg, 95% confidence interval). Make clear which confounders were adjusted for and why they were included **N/A** |
|  |  | (*b*) Report category boundaries when continuous variables were categorized **N/A** |
|  |  | (*c*) If relevant, consider translating estimates of relative risk into absolute risk for a meaningful time period **N/A** |
| Other analyses | 17 | Report other analyses done—eg analyses of subgroups and interactions, and sensitivity analyses **N/A** |
| Discussion | | |
| Key results | 18 | Summarise key results with reference to study objectives **page 11 of revised cleaned version, lines 11-16** |
| Limitations | 19 | Discuss limitations of the study, taking into account sources of potential bias or imprecision. Discuss both direction and magnitude of any potential bias **page 11 of revised cleaned version, lines 18-22** |
| Interpretation | 20 | Give a cautious overall interpretation of results considering objectives, limitations, multiplicity of analyses, results from similar studies, and other relevant evidence **page 12 of revised cleaned version** |
| Generalisability | 21 | Discuss the generalisability (external validity) of the study results **page 12 of revised cleaned version** |
| Other information | | |
| Funding | 22 | Give the source of funding and the role of the funders for the present study and, if applicable, for the original study on which the present article is based **page 13 of revised cleaned version** |

*Give information separately for exposed and unexposed groups.

**Note:** An Explanation and Elaboration article discusses each checklist item and gives methodological background and published examples of transparent reporting. The STROBE checklist is best used in conjunction with this article (freely available on the Web sites of PLoS Medicine at http://www.plosmedicine.org/, Annals of Internal Medicine at http://www.annals.org/, and Epidemiology at http://www.epidem.com/). Information on the STROBE Initiative is available at www.strobe-statement.org.
